# Strength Assessment of Trunk Rotator Muscles: A Multicenter Reliability Study

**DOI:** 10.3390/healthcare11162331

**Published:** 2023-08-18

**Authors:** Angela Rodríguez-Perea, María Dolores Morenas Aguilar, Raquel Escobar-Molina, Darío Martínez-García, Ignacio Chirosa Ríos, Daniel Jerez-Mayorga, Luis Chirosa Ríos, Danica Janicijevic, Waleska Reyes-Ferrada

**Affiliations:** 1 Department of Physical Education and Sport, Faculty of Sports Sciences, University of Granada, 18071 Granada, Spain; arodriguezperea@ugr.es (A.R.-P.); loles_bailen4@hotmail.com (M.D.M.A.); rescobar@ugr.es (R.E.-M.); damaga1991@gmail.com (D.M.-G.); ichirosa@ugr.es (I.C.R.); lchirosa@ugr.es (L.C.R.); 2 Strength & Conditioning Laboratory, CTS-642 Research Group, Department Physical Education and Sports, Faculty of Sport Sciences, University of Granada, 18071 Granada, Spain; 3 Strength & Conditioning Laboratory, CTS-362 Research Group, Department Physical Education and Sports, Faculty of Sport Sciences, University of Granada, 18071 Granada, Spain; jan.danica@gmail.com; 4 Exercise and Rehabilitation Sciences Institute, School of Physical Therapy, Faculty of Rehabilitation Sciences, Universidad Andres Bello, Santiago 7591538, Chile; waleska.reyes@unab.cl; 5 Department of Sports Sciences and Physical Conditioning, Faculty of Education, Universidad Católica de la Santísima Concepción, Concepción 4070129, Chile; 6 Faculty of Health Sciences, Universidad Isabel I, 09003 Burgos, Spain

**Keywords:** core strength, testing, isokinetic, muscle strength dynamometer, reproducibility

## Abstract

Background: Trunk rotator strength plays an important role in sports performance and health. A reliable method to assess these muscles with functional electromechanical dynamometer has not been described. Therefore, the objectives of this paper were (I) to explore the reliability of different strength variables collected in isokinetic and isometric conditions during two trunk rotator exercises, and (II) to determine the relationship of isometric and dynamic strength variables collected in the same exercise. Methods: A repeated measures design was performed to evaluate the reliability of the horizontal cable woodchop (HCW) and low cable woodchop (LCW) exercises. Reliability was assessed using *t*-tests of paired samples for the effect size, the standard error of measurement, the coefficient of variation (CV) and the intraclass correlation coefficient (ICC). The Pearson’s (*r*) correlation coefficient was used to explore the association between isometric and isokinetic tests. Results: HCW exercise is more reliable than LCW exercise in assessing trunk rotator muscles. The strength manifestation that should be used is the average strength, and the most reliable evaluation was the HCW at 0.40 m·s^−1^ concentric (ICC = 0.89; CV = 10.21%) and eccentric (ICC = 0.85; CV = 9.33%) contraction and the dynamic condition that most correlated with the isometric was LWC at 0.50 m·s^−1^ (*r* = 0.83; *p* < 0.01). Conclusion: HCW is a reliable exercise to measure trunk rotator muscles.

## 1. Introduction

Trunk rotators are present in activities of daily living and sports-specific tasks [1]. The relationship between trunk rotator strength (TRS) and sports performance has been studied in sports variables. High levels of strength, power and velocity in trunk rotators improved sports performance such as the speed of throwing the ball in baseball, paddle force in canoe sprinters or swing performance in golfers [2,3,4,5]. In addition, the importance of TRS and the incidence of injuries, such as low back pain and lower limb, have been demonstrated [6,7], showing lower levels of TRS and core stability than in healthy people.

Trunk strength assessment has been carried out in most cases, with isokinetic devices being the gold standard; however, these devices have a limitation in that the type of device establishes the assessment position and sets the straps for the subjects, thereby making the assessment analytical. Several studies evaluated TRS more similarly to sports gestures and without having the participants fixed. These evaluations were performed in half kneeling position [8,9], standing [10] or sitting [11], in tonic mode on a pulley with a trunk rotation exercise (chop and lift and cable woodchop), and high and very high reliability values were obtained.

Performing a test focused on the freedom of movement and the approach to sports gestures is essential to improve sports performance. The trunk rotators are responsible for stabilizing the spine and transferring forces in many sporting motions. However, no specific tests exist to evaluate performance [12]. In a review by Zemkova in 2022, it was concluded that most studies evaluate core strength isometrically or endurance strength; however, very few considered core strength [1]. Furthermore, Kibler et al., 2006, recommends assessing the core dynamically and differentiating the types of contraction that are crucial for sports performance [13].

Currently, new devices such as functional electromechanical dynamometry (FEMD) allow to conduct the assessment of the TRS more specifically to sports gestures and in an isokinetic mode [14]. This device allows one to develop different strength assessment tasks with a cable similar to a pulley where all measured values are recorded. Previous studies have analyzed the reliability of the trunk flexors and extensors with FEMD, providing reliable data for the assessment of this musculature. For both trunk flexors and extensors assessed with FEMD, the lower velocity had a higher reliability and low velocities were most correlated with the isometric assessment [15,16]. In addition, a recent study explored the influence of sex and side dominance on TRS reliability and indicated the importance of sex in the evaluation of trunk rotators; however, no differences in reliability were found according to the dominant side [17]. However, that study did not examine exactly which exercise is more reliable to assess TRS.

Therefore, the objectives in this study were (I) to explore the reliability of different strength variables collected in isokinetic and isometric conditions during two trunk rotator exercises, and (II) to determine the relationship of isometric and dynamic strength variables collected in the same exercise. We hypothesized that the horizontal cable woodchop (HCW) was more reliable than the low cable woodchop (LCW) exercise, the low velocities and isometric contraction were more reliable for assessing trunk strength rotator and the low velocities were more related to the isometric condition.

## 2. Materials and Methods

A repeated measures design was performed to evaluate the reliability of the trunk rotators with FEMD in the HCW and LWC exercises in two centers. All the evaluations were carried out in the Strength and Conditioning Laboratory of the University of University of Granada (Granada, Spain) and the Exercise and Rehabilitation Science Laboratory of the University of Andres Bello (Santiago, Chile). Following a familiarization session, the participants visited the laboratory on two separate occasions, with a 48 h gap between each visit. During these sessions, the subjects performed two exercises utilizing different protocols to evaluate the TRS in both isokinetic and isometric modes.

### 2.1. Participants

Thirty-one physically active university students of Spain (women *n*= 15; men *n* = 16) and twenty-one physically active university students of Chile (women = 7; men *n* = 14) without any musculoskeletal injuries were recruited. The assessment with an FEMD involved three sports scientists (AR-P, WR-F and MDMA); each of the scientists has over eight years’ of experience with this device. A total of 51 participants took part in this study, with the exception of one Spanish woman who withdrew due to a musculoskeletal injury. The descriptive characteristics of the sample are presented in Table 1. Participants were excluded when they had an Oswestry Disability Questionnaire (ODI) score of above 20%, a history of neurological or cardiopulmonary conditions, or abdominal surgery within the last six months. Before providing a written consent to participate, participants were fully informed about the nature, aims and associated risks of the experimental procedure. The study protocol received the approval of the Institutional Review Board of the University of Granada (no. 2560/CEIH/2022) and adhered to the principles described in the Declaration of Helsinki.

### 2.2. Procedures

**Figure 1:**
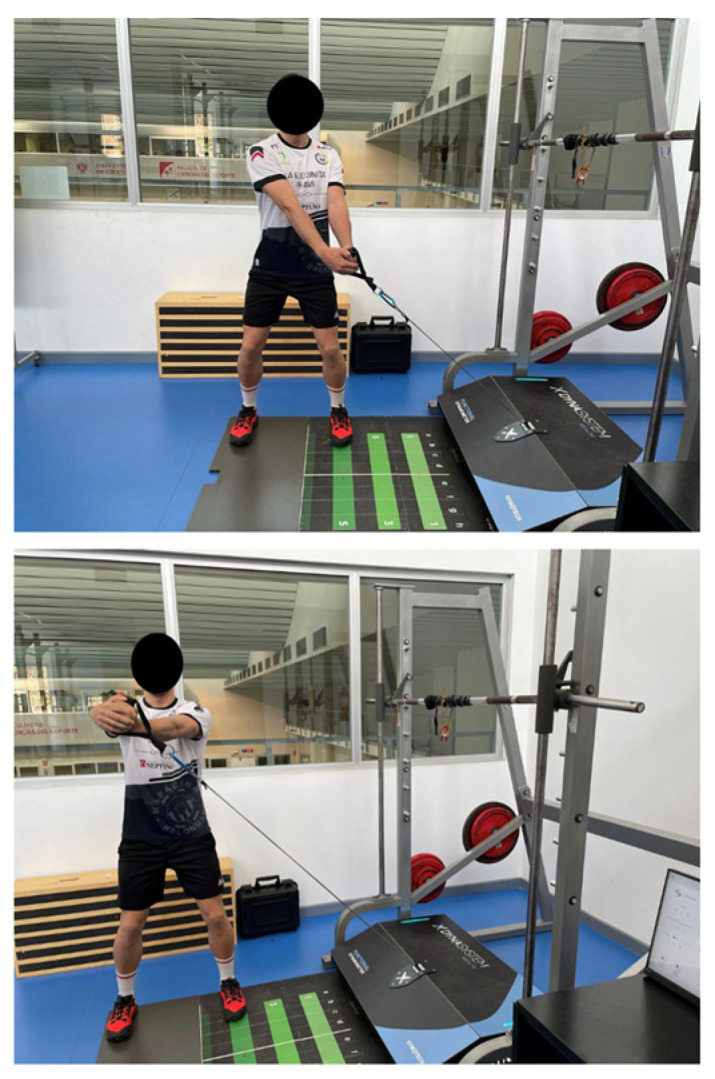
Low cable woodchop exercise.

**Figure 2:**
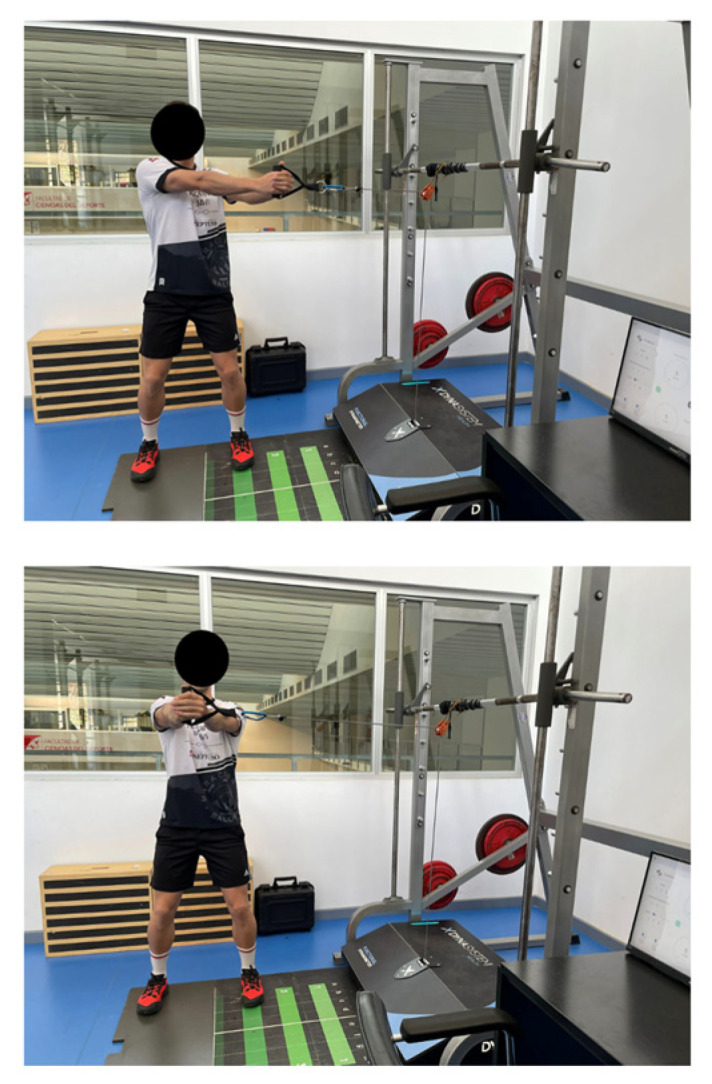
Horizontal cable woodchop exercise.

Isokinetic and isometric strength was evaluated using an FEMD (Dynasystem, Symotech, Spain) [14] with a precision of 3 mm for the displacement, 100 g for the load sensor, a sample rate of 1000 Hz and a speed range of 0.05 ms. In addition, both exercises were performed with a standard grip. The assessment protocol followed was the same as detailed in the article by Rodríguez-Perea et al. (2023) [17]. The performance of the LWC and HCW exercises are shown in Figure 1 and Figure 2, respectively.

A familiarization session with FEMD was attended by the participants. This familiarization was a 60 min session, which started with a 5 min of general and specific mobility, and core activation with prone and glute bridge. Afterward, they performed one set of 10 repetitions for the dominant side of both exercises with an elastic band. Then, the familiarization with FEMD was performed at a velocity of 0.40 m·s^−1^ and 0.60 m·s^−1^ for the HCW exercise and at 0.50 m·s^−1^ and 0.70 m·s^−1^ for the LCW exercise, with subject-specific range of movement (ROM) (1 set of 7 repetitions, 2 submaximal repetitions and 5 maximal). In this session, the ROM of each subject was recorded.

The participants attended under the same conditions as in the familiarization session. The instructions to the participants the same as detailed in the article by Rodríguez-Perea et al. (2023) [17].

### 2.3. Statistical Analysis

#### 2.3.1. Outcome Variables

The three highest repetitions of the average and peak strength for the concentric and eccentric contractions were taken to calculate the dynamic strength. In calculating the isometric strength, the repetition’s peak value and mean value were taken.

#### 2.3.2. Reliability

The descriptive data are presented as mean ± SD. The normality of the data distribution was examined using the Shapiro–Wilk test. Paired sample *t*-tests were conducted to assess the effect size (ES), coefficient of variation (CV), standard error of measurement (SEM), and the intraclass correlation coefficient (ICC) with 95% confidence intervals. Absolute reliability was evaluated through CV and SEM, while relative reliability was assessed using ICC (model 3.1) along with their respective 95% confidence intervals (CI). In order to interpret the magnitude of the ES, a specific scale utilized in training research was employed: negligible (<0.2), small (0.2–0.5), moderate (0.5–0.8), and large (≥0.8) [18]. The magnitude of the intraclass correlation coefficient (ICC) values was assessed using a qualitative scale. According to this scale, ICC values near 0.1 are classified as low reliability, 0.3 as moderate reliability, 0.5 as high reliability, 0.7 as very high reliability, and values approaching 0.9 as extremely high reliability [19]. Bland–Altman analyses were performed to show the level of agreement between tests and retests of LWC and HCW. Heteroscedasticity of errors was also identified in the Bland–Altman plots and defined as a coefficient of determination (*r*^2^) > 0.1. Plots show “the bias” and limits of agreement (LoA) calculated to 95%.

A Pearson correlation coefficient was calculated with a 95% confidence interval for the relation between isometric tests and dynamic tests. The criteria to interpret the magnitude of the r were small (0.10–0.29), moderate (0.30–0.49), large (0.50–0.69), very large (0.70–0.89), extremely large (0.90–1.00). The reliability observed in each evaluation condition was reported using FEMD. Reliability analyses were performed using a customized spreadsheet [20] and the SPSS software package (version 25.0).

Separate analyses were performed for each exercise. In order to interpret the observed magnitude of differences in the coefficients of variation (CV) between the two exercises, the mean CV of all conditions was calculated. A default value of 1.15, representing the smallest important ratio, was utilized for comparison [21].

## 3. Results

**Figure 3:**
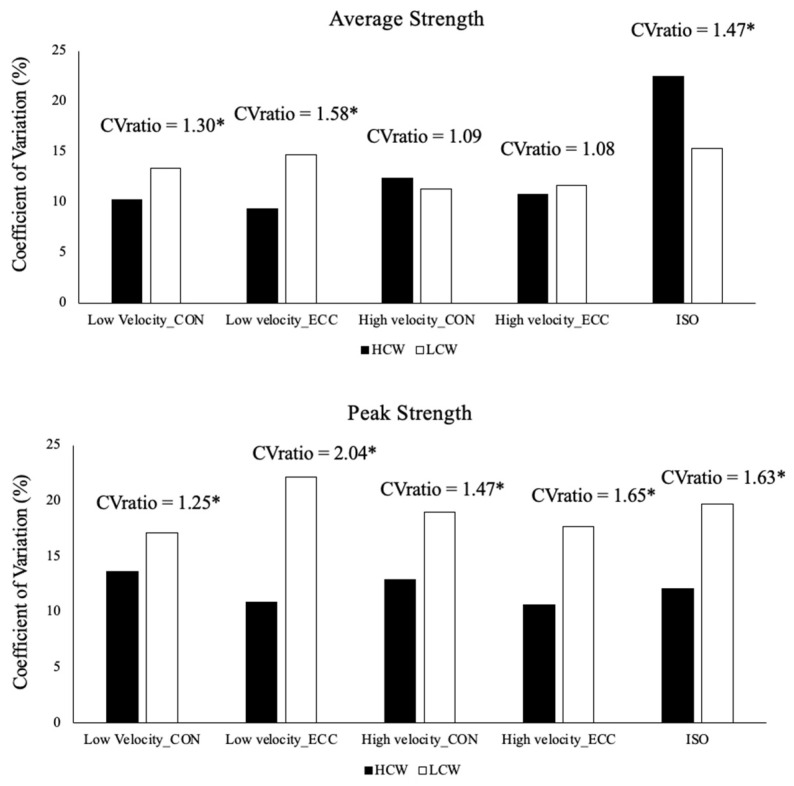
The reliability of the average strength of trunk rotator outcomes between the LCW exercise and HCW exercise was compared (upper panel). Additionally, the reliability of the peak strength of trunk rotator outcomes between LCW exercise and HCW exercise was assessed (lower panel). * Meaningful differences in reliability were determined by examining the CVratio, which is considered significant if it exceeds 1.15. The coefficient of variation (CV) was used as the metric for assessing reliability.

The average and peak strength values of the two exercises combined and the absolute and relative reliability in the test and retest sessions are shown in Table 2. The mean CV was 16.19% and 12.52% of LCW and HCW, respectively. The comparison of the CV through the CVratio revealed that HCW exercises was able to determine the outcomes of the TRS with higher reliability than LCW exercises and the average strength was more reliable than peak strength manifestation in the two exercises (Figure 3). The only exception was high velocity in the average strength which failed to show the differences between the LCW and HCW exercises; it also showed a higher CV when average isometric strength was obtained for LCW exercises as compared to HCW exercises.

**Figure 4:**
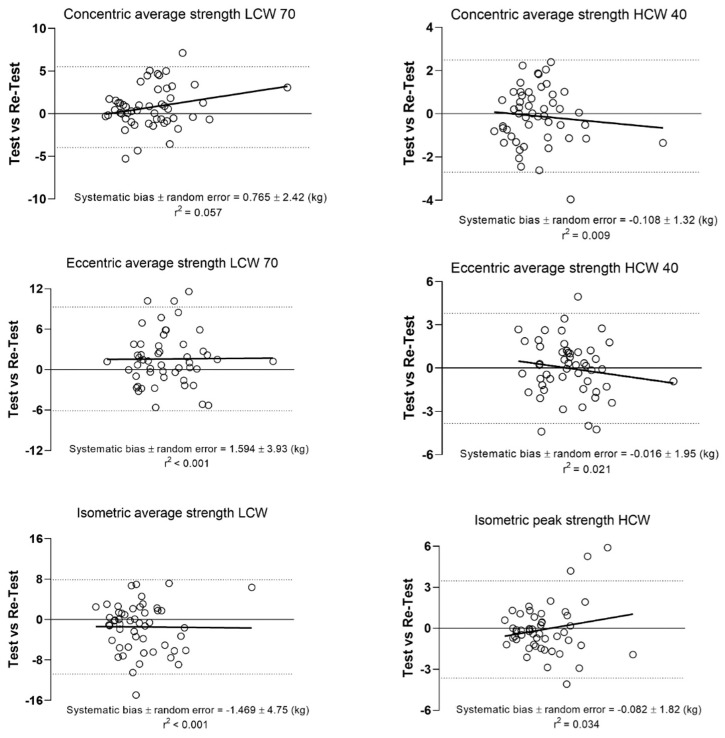
Bland–Altman plots for the measurement of TRS between the test and retest. Each plot depicts the averaged difference and 95% limits of agreement (dashed lines), along with the regression line (solid line).

The most reliable dynamic condition to assess trunk strength rotator was the HCW exercise at 0.40 m·s^−1^ in concentric (ICC = 0.89; CV = 10.21%) and eccentric (ICC = 0.85; CV = 9.33%) contraction and the static condition was the isometric HCW (ICC = 0.80; CV = 12.06%) using average strength. Figure 4 shows the Bland–Altman plots between tests and retests of LWC and HCW of the most reliable condition with the limits of agreement (LOA) and systematics bias. No heteroscedasticity of error was observed (*r* < 0.1) and Bland–Altman plots revealed low random errors (<4.75 kg) for LCW and very low random error (<1.95 kg) for HCW.

The average and peak concentric strength of LWC at 0.50 m·s^−1^ were the best related to the isometric contraction (*r* = 0.83; *p* < 0.01) and the peak eccentric strength of HCW at 0.40 m·s^−1^ (*r* = 0.66; *p* < 0.01) was the best.

## 4. Discussion

The main findings were that HCW exercise is more reliable than LCW exercise in assessing TRS, except for concentric average strength at 0.60 m·s^−1^ and average isometric strength. Therefore, the strength manifestation that should be used is the average strength and the most reliable evaluation was the HCW at 0.40 m·s^−1^ concentric (ICC = 0.89; CV = 10.21%) and eccentric (ICC = 0.85; CV = 9.33%) contraction. Similar results were obtained for trunk flexors and extensors assessed with FEMD, where the highest reliability was obtained at the lowest velocity and average strength, except for the eccentric phase of the flexors, where higher reliability was obtained at intermediate velocities and peak force [15,16]. Moreover, the dynamic condition that most correlated with the isometric was LWC at 0.50 m·s^−1^ (*r* = 0.83; *p* < 0.01). Based on these results, it was found that HCW is a reliable exercise to measure TRS.

The highest strength values were found in the LWC exercise in all conditions. The study’s first hypothesis was confirmed: HCW exercise is more reliable than LCW exercise in assessing TRS. Although the relative reliability values were similar in both exercises (0.62 < ICC > 0.89), HCW exercise obtained better absolute reliability values the LCW exercise with a coefficient of variation values close to 10% in all conditions, except in average isometric strength. In addition, the CV ratio of average strength and peak strength showed that HCW was more reliable in all conditions, except for average strength at the speed of 0.60 m·s^−1^ (CV ratio = 1.08; 1.09) and average isometric strength where the CV was higher in HCW (CV = 22.46%) than LCW (CV = 15.31%).

The average strength should be used to assess TRS with these two exercises. Similar results are found in the study by Zemková et al. [10], which showed that average strength is more reliable than peak strength in assessing strength. However, in many studies that analyze the reliability of TRS, the manifestation taken over the years has been the peak strength, without having studied the behavior of the average strength. In isokinetic devices, the peak strength is almost always taken as a reference [22,23]; however, different studies have analyzed the strength of the trunk with new devices and have shown that the most reliable manifestation is the average strength [15,16].

In line with our second hypothesis, the most reliable evaluation was HCW at 0.40 m·s^−1^. Comparing these reliability results is difficult due to the novelty of both the test and the device. Regarding the evaluation of TRS in isokinetic devices, these results agree with previous studies where the TRS was evaluated at two isokinetic velocities, obtaining similar reliability values (ICC = 0.80–0.90; CV = 11–17%), showing better absolute and relative reliability values to the lowest velocity (60°) [22]. In another study that evaluated TRS in an isometric mode, the coefficient of variation value was 3.5–4.5% [24]. Another way to assess the TRS is with portable dynamometers and without other material, in a lying face-up position, which can obtain high-reliability values (ICC = 0.80) [25], or with pulleys in a dynamic mode in a seated position (ICC = 0.94–0.97) or half kneeling position (ICC = 0.83–0.97) [9]. Trunk rotators have also been evaluated with a linear position transducer in half kneeling position and with a pulley (CV = 7.4–16.3%; ICC 0.54–0.83) [8]. One study analyzed trunk rotator isometric strength very specifically for sprint flat-water kayakers with a load cell and obtained very high reliability values (ICC 0.98–0.99; CV 3.7–4.0%) [26]. The study most similar to our research was the one carried out by Zemkova et al. [10]. They evaluated standing cable wood chop exercises using a pulley and a portable dynamometer with different weights, obtaining very high reliability values (ICC = 0.93–0.97).

To our knowledge, no study related maximal dynamic strength to maximal isometric strength of the trunk rotators. However, two studies evaluated trunk flexor and extensor strength with FEMD and analyzed the relationship between dynamic and isometric strength. In the case of the trunk flexors, the lowest velocity and the peak strength was the one that most correlated in the concentric phase and the highest velocity in the eccentric phase and the trunk extensors; the opposite occurred in extensor strength, where the mean strength velocity for the concentric phase was most closely related to the isometric phase and the eccentric with peak strength had the lowest velocity [15,16]. In the TRS, the dynamic condition that most correlated with the isometric was LWC at 0.50 m·s^−1^ in the concentric phase.

In recent reviews and meta-analyses on associations between measures of TRS and physical performance, small effect sizes for TRS have been shown to improve muscular strength, muscular power, balance, and athletic performance. However, none of the studies included in the meta-analysis evaluated the strength of the trunk in a similar way to the task of the sports, but rather majority of them evaluated the strength of the trunk endurance and only one evaluated the TRS, knowing the implication that this musculature has in many sports gestures [27].

This is the first study that assesses the reliability of TRS with two exercises frequently performed during athlete’s training in both isokinetic and isometric modes. Knowing how reliable these exercises are in assessing the TRS helps to improve the evaluations of this musculature in a more similar way to sports tasks. Carrying out the study in a multicentric way has allowed us to increase the sample size and verify that the test is reliable regardless of the person who assesses TRS. The advantage of using this device is that it allows us to evaluate the TRS in a freer way; in addition, only this device is needed, which is easy to use, affordable and transportable. Previous studies used isokinetic devices, which are not mobile and are expensive, or more than one device such as a pulley and a load cell or a pulley and a dynamometer.

In practical implementation, assessing strength without the subject being attached to a device and seated allows us to evaluate the TRS in a way that is more similar to sports gestures or activities of daily living. In addition, through the test’s coefficient of variation the test we can understand the effects of special training or rehabilitation programs. On the other hand, a reliable evaluation protocol has been created with FEMD that allows us to know the best velocity and exercise to evaluate this musculature. All the assessed protocols have shown reliable values, so if the gesture is more similar to the LWC exercise, it could be used equally. In the clinical field, it is recommended to use the most reliable protocol; however, in sports, it is recommended to use the velocity and exercise closest to the sport performed. In addition, when studying the reliability of an exercise performed in sports training in an isokinetic mode, this exercise could also be used to establish training loads or reference velocities.

The limitations of our study were that only healthy and young subjects were evaluated, so the data cannot be extrapolated to other populations, such as older adults or people with back pain. Furthermore, despite obtaining good reliability results, the familiarization process was short. It could be more affected in the eccentric phase of the movement, where lower reliability values were obtained when the average strength was used. In addition, there was a learning effect with some effect sizes greater than 20% in the LWC exercise. In future research, it could be verified if the reliability is affected by the level of training experience of the subjects and using a free range of movement.

## 5. Conclusions

The results obtained in our study indicate that HCW exercise is more reliable in assessing TRS with FEMD in young healthy subjects. Average strength at 0.40 m·s^−1^ is the most reliable condition to assess HCW, with an ICC value of 0.89. Moreover, the dynamic condition that most correlated with the isometric assessment was LWC exercise at 0.50 m·s^−1^.

## Figures and Tables

**Table 1:** Characteristics of the sample.

	Total (*n* = 51)	Center 1 (*n* = 30)	Center 2 (*n* = 21)
Age (years)	22.39 (3.5)	22.40 (4.5)	22.38 (1.3)
Height (m)	1.72 (0.08)	1.72 (0.8)	1.71 (0.9)
Weight (kg)	73.01 (12.8)	71.97 (11.0)	74.5 (15.1)
BMI (kg/m^2^)	24.73 (3.3)	24.30 (2.4)	25.3 (4.3)
OLBPD (%)	2.71 (4.7)	3.27 (4.9)	1.9 (4.3)
ROM_LWC (cm)	75.2 (4.3)	74.5 (4.0)	76.2 (4.6)
ROM_HWC (cm)	65.5 (3.9)	65.1 (3.5)	66.3 (4.4)

Data are presented as mean (standard deviation). BMI = body mass index; OLBPD = Oswestry low back pain disability; ROM = range of movement; HWC = horizontal cable woodchop; LWC = low cable woodchop.

**Table 2:** Absolute and relative test–retest reliability of the average and peak strength of LCW and HCW exercises.

		Test (Kg)	Retest (Kg)	*p*-Value	ES	ICC (95% CI)	CV (95% CI)	SEM (95% CI)
Average Strength								
LCW_0.50 m·s^−1^	Con	15.9 (6.0)	15.0 (4.4)	0.04	−0.16	0.85 (0.75–0.91)	13.32 (11.14–16.56)	2.06 (1.72–2.56)
	Ecc	23.0 (7.8)	22.7 (5.6)	0.64	−0.05	0.76 (0.62–0.86)	14.73 (12.32–18.30)	3.37 (2.82–4.19)
LCW_0.70 m·s^−1^	Con	15.5 (5.4)	14.7 (4.9)	0.03	−0.15	0.89 (0.82–0.94)	11.32 (9.47–14.08)	1.71 (1.43–2.12)
	Ecc	24.6 (6.3)	23.0 (6.3)	0.01	−0.25	0.81 (0.69–0.89)	11.69 (9.78–14.53)	2.78 (2.32–3.45)
LCW_ISO	Iso	21.2 (7.1)	22.7 (7.2)	0.03	0.21	0.78 (0.65–0.87)	15.31 (12.81–19.03)	3.36 (2.81–4.18)
HCW_0.40 m·s^−1^	Con	9.1 (2.8)	9.2 (2.9)	0.57	0.04	0.89 (0.82–0.94)	10.21 (8.54–12.69)	0.94 (0.78–1.16)
	Ecc	14.8 (3.3)	14.8 (3.6)	0.95	0.00	0.85 (0.75–0.91)	9.33 (7.80–11.59)	1.38 (1.15–1.71)
HCW_0.60 m·s^−1^	Con	8.9 (3.0)	8.9 (2.9)	0.89	−0.01	0.86 (0.77–0.92)	12.33 (10.32–15.33)	1.10 (0.92–1.37)
	Ecc	15.1 (3.5)	15.0 (3.5)	0.71	−0.03	0.79 (0.66–0.88)	10.78 (9.02–13.40)	1.62 (1.36–2.02)
HCW_Iso	Iso	9.7 (3.9)	9.5 (2.2)	0.69	−0.05	0.54 (0.31–0.71)	22.46 (18.79–27.92)	2.16 (1.81–2.69)
Peak Strength								
LCW_0.50 m·s^−1^	Con	25.1 (8.9)	25.1 (9.0)	0.93	−0.01	0.78 (0.64–0.87)	17.07 (14.28–21.22)	4.28 (3.58–5.33)
	Ecc	40.4 (15.4)	38.4 (12.3)	0.26	−0.14	0.62 (0.41–0.76)	22.14 (18.52–27.52)	8.72 (7.30–10.84)
LCW_0.70 m·s^−1^	Con	26.2 (10.7)	24.0 (8.3)	0.02	−0.24	0.76 (0.62–0.86)	18.96 (15.86–23.57)	4.76 (3.99–5.92)
	Ecc	44.8 (16.1)	40.5 (13.3)	0.01	−0.29	0.75 (0.60–0.85)	17.64 (14.76–21.93)	7.53 (6.30–9.36)
LCW_ISO	Iso	25.0 (9.9)	26.1 (8.7)	0.25	0.12	0.72 (0.55–0.85)	19.67 (16.45–24.45)	5.03 (4.21–6.25)
HCW_0.40 m·s^−1^	Con	14.7 (3.2)	14.6 (3.4)	0.94	−0.01	0.64 (0.44–0.78)	13.66 (11.43–16.98)	2.00 (1.68–2.49)
	Ecc	19.4 (3.9)	19.7 (4.3)	0.62	0.05	0.74 (0.58–0.84)	10.84 (9.07–13.48)	2.12 (1.77–2.63)
HCW_0.60 m·s^−1^	Con	16.1 (3.8)	16.1 (3.8)	1.00	0.00	0.71 (0.54–0.82)	12.90 (10.79–16.03)	2.07 (1.74–2.58)
	Ecc	21.5 (5.0)	20.8 (4.5)	0.15	−0.14	0.78 (0.65–0.87)	10.66 (8.92–13.26)	2.26 (1.89–2.81)
HCW_Iso	Iso	10.6 (3.0)	10.7 (2.7)	0.75	0.03	0.80 (0.68–0.88)	12.06 (10.09–14.99)	1.28 (1.07–1.60)

Data are as presented as mean (SD). LWC = low cable woodchop; HCW = horizontal cable woodchop; ISO = isometric contraction; CON = concentric contraction; ECC = eccentric contraction; CV = coefficient of variation; SEM = standard error of measurement (kg); ICC = intraclass correlation coefficient; 95% CI = 95% confidence interval.

## Data Availability

The data that support the findings of this study are available on reasonable request from the corresponding author. The data are not publicly available due to privacy or ethical restrictions.
